# A Novel E2F1-EP300-VMP1 Pathway Mediates Gemcitabine-Induced Autophagy in Pancreatic Cancer Cells Carrying Oncogenic KRAS

**DOI:** 10.3389/fendo.2020.00411

**Published:** 2020-06-23

**Authors:** Alejandro Ropolo, Cintia Catrinacio, Felipe Javier Renna, Veronica Boggio, Tamara Orquera, Claudio D. Gonzalez, Maria I. Vaccaro

**Affiliations:** ^1^Department of Pathophysiology, Institute of Biochemistry and Molecular Medicine (UBA-CONICET), School of Pharmacy and Biochemistry, University of Buenos Aires, Buenos Aires, Argentina; ^2^CEMIC University Institute, Buenos Aires, Argentina

**Keywords:** pancreatic cancer, gemcitabine, autophagy, VMP1, E2F1

## Abstract

Autophagy is an evolutionarily preserved degradation process of cytoplasmic cellular constituents, which participates in cell response to disease. We previously characterized VMP1 (Vacuole Membrane Protein 1) as an essential autophagy related protein that mediates autophagy in pancreatic diseases. We also demonstrated that VMP1-mediated autophagy is induced by HIF-1A (hypoxia inducible factor 1 subunit alpha) in colon-cancer tumor cell lines, conferring resistance to photodynamic treatment. Here we identify a new molecular pathway, mediated by VMP1, by which gemcitabine is able to trigger autophagy in human pancreatic tumor cell lines. We demonstrated that gemcitabine requires the VMP1 expression to induce autophagy in the highly resistant pancreatic cancer cells PANC-1 and MIAPaCa-2 that carry activated *KRAS*. E2F1 is a transcription factor that is regulated by the retinoblastoma pathway. We found that E2F1 is an effector of gemcitabine-induced autophagy and regulates the expression and promoter activity of VMP1. Chromatin immunoprecipitation assays demonstrated that E2F1 binds to the *VMP1* promoter in PANC-1 cells. We have also identified the histone acetyltransferase EP300 as a modulator of VMP1 promoter activity. Our data showed that the E2F1-EP300 activator/co-activator complex is part of the regulatory pathway controlling the expression and promoter activity of VMP1 triggered by gemcitabine in PANC-1 cells. Finally, we found that neither VMP1 nor E2F1 are induced by gemcitabine treatment in BxPC-3 cells, which do not carry oncogenic KRAS and are sensitive to chemotherapy. In conclusion, we have identified the E2F1-EP300-VMP1 pathway that mediates gemcitabine-induced autophagy in pancreatic cancer cells. These results strongly support that VMP1-mediated autophagy may integrate the complex network of events involved in pancreatic ductal adenocarcinoma chemo-resistance. Our experimental findings point at E2F1 and VMP1 as novel potential therapeutic targets in precise treatment strategies for pancreatic cancer.

## Introduction

Pancreatic ductal adenocarcinoma (PDAC) is one of the most aggressive human malignancies with 8–9% 5-year survival rate (1). Despite the progress in the knowledge of the disease, it remains a calamitous neoplasia. Up to 60% of patients have advanced pancreatic cancer at the time of diagnosis, and their median survival time is 3–6 months (2). Its poor prognosis has been attributed to a tendency regarding early vascular dissemination and spreading to regional lymph nodes, and to the incapacity to make a diagnosis while the tumor is still surgically removable (2). This is caused both by the aggressive nature of the disease, the lack of specific symptoms and early detection tools, and the refractory response to traditional cytotoxic agents and radiotherapy (3, 4). Furthermore, pancreatic cancer cells get more malignant and survive with an extremely low blood supply (2, 3). Up to now, contradictory data are available concerning autophagy activity and its regulation by specific autophagy related (ATG) proteins in pancreatic cancer cells. Experimental evidence places autophagy as a mechanism for survival of tumor cell under adverse environmental conditions, or as a defective mechanism of programmed cell death that promotes pancreatic cancer cell resistance to treatment (5–7).

At present, the first option for resectable tumors in pancreatic cancer is adjuvant chemotherapy before surgical resection (8, 9). However, most patients are in an advanced stage at the time of diagnosis and in these cases, chemotherapy is used as the first option. Regarding chemotherapy, gemcitabine used alone or in combination with nabpaclitaxel represent one of the most effective therapy (9, 10), despite its poor efficacy in terms of overall patient survival (11). Gemcitabine works by causing apoptosis of malignant cells in pancreatic cancer (12, 13). Intrinsic and acquired factors are involved in gemcitabine resistance. Several of them related to the transport and metabolism of gemcitabine (14) and associated with the tumor microenvironment, among others (15, 16).

Interestingly, recent studies highlight the importance of autophagic flux in acquiring resistance to gemcitabine in pancreatic cancer tumor cells (17–19). Macroautophagy (hereafter autophagy), is an evolutionarily conserved process that involves the sequestration and delivery of cytoplasmic components into the lysosome, where they are degraded and recycled (20). Autophagy is involved in the turnover of long-lived proteins and other cellular macromolecules. It has also been involved in the physiological responses to exercise and aging and is implicated in different pathophysiological processes such as neurodegenerative disorders, cardiovascular, pulmonary diseases, and cancer (21–24). Autophagy correlates with poor patient outcome in pancreatic cancers (25), and it has been suggested that autophagy is required for tumor growth (6, 7). For instance, in mice with pancreas containing an activated oncogenic allele of *KRAS* proto-oncogene, GTPase (KRAS), the most frequent mutation in PDAC (26), a small number of pre-cancerous lesions are developed that become PDAC randomly over time (27). KRAS activates the expression of the Vacuole Membrane Protein 1 (VMP1) to induce and maintain autophagy levels in pancreatic tumor cells (28). Accordingly, mice lacking the essential autophagy genes ATG5 or ATG7 acquire pre-invasive low-grade pancreatic intraepithelial neoplasia lesions, but progression to high-grade pancreatic intraepithelial neoplasia lesions and PDAC is blocked (27). This evidence highlight the relevance of KRAS-induced autophagy in the malignant transformation of pancreatic tumor cells.

Autophagy involves the formation of double-membrane structure, autophagosomes, around the cellular components targeted for degradation, which include large structures such as organelles and protein aggregates (29). Autophagy is mediated by a set of evolutionarily conserved gene products (termed the ATG proteins) originally discovered in yeast (30). In mammalian cells, the sequential association of at least a subset of the ATG proteins, referred to as the core molecular machinery (29), leads to the autophagosome formation. VMP1 belongs to these essential ATG proteins. We have demonstrated that VMP1 expression triggers autophagy in mammalian cells even under nutrient-rich conditions (31, 32). By contrast, autophagy is completely blocked in the absence of VMP1 expression (31). VMP1 autophagy-related function requires its hydrophilic C-terminal domain of 20 amino acids (VMP1-ATGD) (32). This domain binds directly to the Bcl-2 binding domain (BH3) motif of beclin 1 (BECN1) leading to the formation of a VMP1-BECN1-PI3KC3 (phosphatidylinositol 3-kinase catalytic subunit type 3) complex at the site where autophagosomes are generated (33, 34).

VMP1 is not expressed in normal pancreas, however its expression is early activated in pancreas suffering experimental diabetes mellitus, experimental and human pancreatitis, and in human pancreatic cancer cells (35–39). Interestingly, VMP1 prevents pancreatic cell death induced by acute pancreatitis (35). In previous studies, we found that VMP1 expression is induced by mutated KRAS in pancreatic tumor cells (28). KRAS is a member of the Ras family of GTP-binding proteins that mediate a wide variety of cellular functions including proliferation, differentiation, and survival. KRAS mutation is one of the earliest genetic events in human PDAC (40). Besides, it has been demonstrated that VMP1 down-regulation reduces cell resistance of pancreatic cells to chemotherapeutic drugs as Imatinib, Cisplatin, Adriamycin, Staurosporin, and Rapamycin (41). In colon cancer cells, we have recently shown that the HIF-1A-VMP1 autophagic pathway is involved in the resistance to photodynamic therapy in colon cancer cells (42). Therefore, we hypothesized that VMP1 is involved in the tumor cell response to chemotherapy in pancreatic cancer cells.

Here, we study the role of autophagy and its molecular mechanism involved in the pancreatic tumor cell response to chemotherapy. We identified a new regulatory pathway, which is activated in high resistant pancreatic tumor cells, carrying oncogenic KRAS, under gemcitabine treatment but not in sensitive cells to chemotherapy. This molecular mechanism includes the activation of E2F transcription factor 1 (E2F1) that binds to VMP1 promoter to enhance VMP1-mediated autophagy. We also identified the histone acetyltransferase EP300 (E1A binding protein p300), as a modulator of this promoter activity. Our data show that the E2F1-EP300 activator/co-activator complex is part of the regulatory pathway controlling VMP1 expression triggered by gemcitabine. Together these data point at E2F1 as a regulatory factor modulating VMP1-mediated autophagy in human pancreatic cancer cells and integrate this degradative cellular process into the complex network of events involved in PDAC chemoresistance.

## Materials and Methods

### Mammalian Cell Lines, Transfections, and Treatments

Human pancreatic cancer cell lines with mutated KRAS, PANC-1 (KRASG12D), and MIAPaCa-2 (KRASG12C), and human pancreatic cancer cell line with wild type *KRAS*, BxPC-3 (43), and also a human HeLa cell line were obtained from American Type Culture Collection. PANC-1, MIAPaCa-2, and HeLa cells were cultured in Dulbecco's modified Eagle's medium (Biological Industries) containing 10% fetal bovine serum (Natocor). BxPC-3 cells were cultured in RPMI 1640 medium (Biological Industries) containing 10% fetal bovine serum (Natocor). All cell culture mediums were supplemented with 100 U μl^−1^ penicillin, and 100 μg μl^−1^ streptomycin (Biological Industries). All cell lines were maintained at 37°C under a humidified atmosphere with 5% CO_2_. Mycoplasma contamination is periodically checked by PCR, each time a cell line enters the laboratory, and then monthly for each cell line currently in use. Cells were seeded 24 h before transfection and treatments to reach a 60% confluence. Cells were transfected using FuGENE-6 Transfection Reagent (Promega) as indicated by the manufacturer. Gemcitabine (Elli Lilly) and chloroquine (Sigma-Aldrich) were prepared according to the manufacturer's instructions. Cells were treated with 20 μM gemcitabine (Elli Lilly) and/or 10 μM chloroquine (Sigma) for different times when appropriate.

### Expression Vectors

Plasmid pRFP-LC3 (microtubule-associated protein 1 light chain 3 fused to red fluorescent protein) was kindly provided by Dr. Maria I. Colombo [Universidad Nacional de Cuyo, Consejo Nacional de Investigaciones Científicas y Técnicas (CONICET), Argentina]. The vector pEGFP-VMP1 (enhanced green fluorescent protein fused to VMP1) was designed and constructed as previously reported (31). Expression vector for E2F1 was kindly provided by Dr. Cánepa (Facultad de Ciencias Exactas y Naturales, Universidad de Buenos Aires, Argentina). The expression vector Flag-EP300 was kindly provided by Dr. Donald Tindall (Mayo Clinic, Rochester, MN, USA). The shRNA targeting EP300 (shEP300) was obtained from Sigma-Aldrich (St. Louis, MO). VMP1 and E2F1 down-regulation was made using the plasmid pCMS3-1p-EGFP, which was kindly provided by Dr D. Billadeau (Department of Immunology, College of Medicine, Mayo Clinic, USA), and contains a separate transcriptional cassette for EGFP to identify transfected cells. A plasmid contains an VMP1 short hairpin RNA (shVMP1) construction (sense 5′-GGCAGAAUAUUGUCCUGUG-3′, and antisense 5′-CACAGGACAAUAUUCUCUGCC-3′) for VMP1 down-regulation. Another plasmid contains an E2F1 short hairpin RNA (shE2F1) construction (sense 5′-GACGTGTCAGGACCTTCGT-3′, and antisense 5′-CTGCACAGTCCTGGAAGCA-3′) for E2F1 down-regulation. A vector with a scramble sequence was used as a control short hairpin RNA (shControl).

### Cell Viability Assay by Trypan Blue Method

Cells were seeded into 6-well-plates at 1 × 10^5^ cells per well with 3 ml growth medium. The following day, PANC-1, MIAPaCA-2, and BxPC-3 cells were treated with 20, 200, or 2,000 μM gemcitabine for 24, 48, and 72 h. The number of viable cells was then determined by the Trypan blue method. Cells were raised by gentle pipetting into the growth medium, and 0.5 ml of the cell suspension was taken and mixed with 0.5 ml of a Trypan Blue 0.2% w/v in phosphate-buffered saline (PBS) solution and incubated for 3 min at room temperature. Colored (dead) and non-colored (live) cells were counted in a Neubauer chamber. The percentage of viable cells under gemcitabine treatment regarding to control was determined according to the following formula: % Viable cells = NT/NC × 100, where NC is the number of viable cells in the control and NT is the number of viable cells under treatment at the same time of incubation.

### RNA Extraction and Quantitative Real-Time PCR (qPCR)

Cells lines were grown in 6-well-plates at 3.5 × 10^5^ cells per well with 3 ml growth medium. After the corresponding transfections and treatments, RNA extraction from cell cultures was performed with the TRIzol reagent (Invitrogen). The concentration of RNA was determined by Vision Life Science Spectrophotometer (Hoefer) and RNA was running in an agarose gel to check its quality. RNA purified (2 μg) were used and treated with 1 μl of RNase-free DNase I (Invitrogen) in a final volume of 10 μl containing 1X DNAse (deoxyribonuclease) buffer. The 10 μl of DNAse treatment was incubated with 100 nM random primers (N6) at 70°C for 5 min. Then, 1X MMLV buffer, dNTPs (deoxynucleoside triphosphates) to 1 mM and 1 μl of the enzyme MMLV reverse transcriptase (Promega) were added in a final volume of 20 μl, incubated at 25°C for 5 min and then at 37°C for 1 h (RT). The RT-PCR reaction was performed on a Techne cycler. For the qPCR, the primers described below were used with 0.5 μl of RT per reaction in a final volume of 25 μl using the Master Mix Real Mix (Biodynamics). The qPCR was performed on RG 6000 cycler (Corvette). The cycling conditions were as follows: 95°C for 2 min followed by 40 cycles of 95°C for 20 s, 58°C for 20 s, and 72°C for 30 s. Transcript level of VMP1 mRNA was normalized by comparison with mRNA of β-actin and was calculated using the 2^−ΔΔ*CT*^ method (44, 45). Specific primers were used for VMP1: forward 5′-GGTGCTGAACCAGATGATGA-3′ and reverse, 5′-GCACCAAAGAAGGTCCAAA-3′; and for β-actin: forward 5′-GACTTCGAGCAAGAGATGG-3′ and reverse, 5′-GCACTGTGTTGGCGTACAG-3′.

### Western Blot Analysis

Cells were seeded into 60 mm culture dishes at 8 × 10^5^ cells per dish with 5 ml growth medium. After different treatments and transfections, cells were lysed in ice-old RIPA buffer (150 mM NaCl, 1% Triton X-100, 1% sodium deoxycholate, 0.1% SDS, 10 mM Tris-HCl pH 7.2, 5 mM EDTA) containing Phosphatase and Protease Inhibitor Cocktail (Sigma–Aldrich). Protein concentration was determined using the bicinchoninic acid (BCA) protein assay reagent (Pierce). Equal amount of protein was analyzed on SDS-PAGE and transferred to polyvinylidene fluoride PVDF membranes (0.22 μm pore size, Millipore). The membranes were blocked with Odyssey Blocking Buffer (LI-COR) at room temperature for 1 h and incubated with the corresponding primary antibodies overnight at 4°C. The primary antibodies used were anti-VMP1 (1:1,000; rabbit mAb #12929, Cell Signaling Technology), anti-E2F1 (1:500; mouse mAb CS204394; Millipore), anti-Actin (1:4,000; rabbit polyAb A2066; Sigma–Aldrich). After incubation, the membrane was washed four times with PBS containing 0.1% Tween-20 (PBST) and twice with PBS, and then incubated with the corresponding IRDye secondary antibody (1:15,000, IRDye® 680LT Goat anti-Rabbit IgG or 1:10,000, IRDye® 800CW Goat anti-Mouse IgG, LI-COR) in Odyssey Blocking Buffer (LI-COR) for 2 h at room temperature. After, the membrane was washed four times with PBST, twice with PBS, and scanned with Odyssey® SA (LI-COR). For LC3 western blotting, the membranes were blocked with 5% (w/v) non-fat dry milk in TRIS-buffered saline (TBS) containing 0.1% Tween-20 (TBST) at room temperature for 1 h and incubated with the primary antibody anti-LC3B (1:1,000, Rabbit mAb #3868, Cell Signaling Technology) overnight at 4°C. After the incubation, the membrane was washed four times with TBST and twice with TBS, then incubated with anti-rabbit HRP-conjugated (1:3,000, Amersham NA934, GE Healthcare) secondary antibody in TBST with 5% (w/v) non-fat milk for 2 h at room temperature. Next, the membrane was washed four times with TBST and twice with TBS and incubated with PIERCE ELC Plus Western blotting Substrate (Cat# 32134, Thermo Scientific) according to manufacturer's instructions. Finally, the membrane was scanned with cDigit Blot Scanner (LI-COR). We used ImageJ software to determine protein bands density. Relative densitometry normalized to actin is expressed as the mean ± SD of three different experiments.

### Fluorescence Microscopy

To determine autophagy, cells were growing on glass slides into 24-well-plates. They were seeded at 5 × 10^4^ cells per well with 1 ml growth medium. Twenty-four hours later, cells were co-transfected with a red fluorescent protein fused to LC3 (RFP-LC3) expression vector and the indicated plasmid, and then treated with gemcitabine. Next, cells were fixed with 4% p-formaldehyde in PBS for 15 min, and immediately washed several times with PBS. Samples were mounted in DABCO (Sigma–Aldrich) and observed using a fluorescence microscope Nikon Eclipse 200 (Plan100), or an inverted LSM Olympus FV1000 using an UPLSAPO 60X O NA: 1.35 objective. We consider a cell positive for autophagy when RFP-LC3 has a punctate staining and not diffused protein remains. The number of fluorescent cells with punctate staining per 100 fluorescent RFP-LC3 transfected cells was determined in three independent experiments. To quantify, the number of fluorescent cells with punctate staining was counted in six random fields representing 100 fluorescent cells and expressed as the mean ± SD of combined results.

### *In silico* Analysis

Genomic details and characteristics from human VMP1 gene were collected from the Ensembl Genome database. VMP1 promoter prediction was done using the Gene2Promoter utility; transcription factors binding sites and additional information was obtained using RegionMiner, MatBase, and MatInspector tools (Genomatix Software). Supporting evidence was found using the Neural Network Promoter Prediction program (BDGP version 2.2) and FPROM (Softberry). Alibaba 2.1 and PROMO 3.0 were also used for transcription factors' consensus sequences search.

### Cloning of *VMP1* Promoter Into Reporter Vector

Genomic DNA from HeLa cells was extracted using TRIzol reagent (Invitrogen). PCR was performed to amplify a fragment of 3,005 bp and then three progressively shorter ones of 1,977, 1,469, and 883 bp from the 5′ upstream region of *VMP1* using specific primers and HeLa DNA as template. PCR was done in a final volume of 50 ml as follows: 1× Pfu DNA Polymerase Buffer (with MgSO4), 300 mM dNTPs (deoxynucleosyde triphosphates), 0.4 mM Primers sense/anti-sense, 50 ng genomic DNA, 1.5U Pfu DNA Polymerase (Promega). Amplification was performed according to an initial denaturation step of 1.5 min at 94°C, followed by 30 cycles at 94°C for 30 s, 59°C for 30 s, 73°C for 8 min, and a final step at 73°C for 5 min. PCR products were run in agarose gels and amplicons were recovered with a Gel Band Purification Kit (GE Healthcare). Primers had restriction sites for SalI (Forward) and HindIII (Reverse) enzymes for subsequent cloning of the fragments in pGemT easy vector (Promega). Amplicons integrity was confirmed by sequence analysis (MacroGen). All constructs were then subcloned in the luciferase reporter pGL3 Basic Vector (Promega), with SalI and HindIII enzymes. As a result, the constructs pGL3.vmp1-883, pGL3.vmp1-1469, pGL3.vmp1-1977, and pGL3.vmp1-3005 were obtained.

### Luciferase Reporter Assays

For luciferase assays cells were plated 24 h before transfection in 12-well-plates at 1.4 × 10^5^ cells per well. Cells were used at 60% confluence for pGL3.vmp1 promoter constructs transfection with FuGENE6 Transfection Reagent (Promega). Ratio used in each case was 1.5 ml FuGENE6 per 1 mg DNA. When two plasmids were transfected, we used 0.4 mg of pGL3 reporter and 0.6 mg of expression vector. In shRNA assays we used 1.5 mg of shRNA and 0.5 mg pGL3 reporter vectors. Treatments were done 24 h after transfection. In co-transfection experiments with expression vectors, cells were processed 48 h after transfection. Each condition was tested in triplicate. Cells were washed with cold PBS and lysed with 100 ml of 1× Cell Culture Lysis Buffer (Promega). 96-well-plates were used for activity assays. In each well 40 μl of luciferase substrate was added to 20 μl of lysate and a 5-s reading was done for luminescence measurement in a Victor3 1420 multilabel counter (PerkinElmer). Results were normalized to protein concentration. Relative light units (RLU) value was calculated as luciferase activity/protein concentration.

### Chromatin Immunoprecipitation (ChIP) Assay

Chromatin immunoprecipitation was conducted following the Pierce Agarose Chip kit (Thermo Scientific). Briefly, PANC-1 cells were culture into 100 mm culture dishes at 3 × 10^6^ cells per dish with 13 ml growth medium. The next day, cells were treated with 20 μM gemcitabine and 24 h were cross-linked with 1% formaldehyde directly into the media for 10 min at room temperature. The cells were then washed and scraped with PBS and collected by centrifugation at 800 × g for 5 min at 4°C, resuspended in cell lysis buffer and incubated on ice for 15 min. The pellet was then resuspended in nuclear lysis buffer and sheared to fragment DNA to about 700 bp. Samples were then immunoprecipitated using a E2F1 antibody or normal rabbit IgG (Millipore) overnight at 4°C on a rotating wheel. Following immunoprecipitation, samples were washed and eluted using the chromatin immunoprecipitation kit in accordance with the manufacturer's instructions. Cross-links were removed at 62°C for 2 h followed by 10 min at 95°C and immunoprecipitated DNA was purified and subsequently amplified by PCR. PCR was performed using seven primer sets for the seven areas containing potential E2F1 binding sites in the VMP1 promoter sequence: forward, 5′-GCATCTCACTTTGTCACCCAG-3′ and reverse, 5′-ACTTGAGGTCAGGAGTTCGAGAC-3′; (2) forward, 5′-CAGGCTGTTCTCAAACTCCTGG-3′ and reverse, 5′-GCACCATACTAGACTCTGGGA; (3) forward, 5′-TCCCAGAGTCTAGTATGGTGC-3′ and reverse, 5′-CGATATCGCTCCATTGCTCTCCA-3′; (4) forward, 5′-GAGTAGCTGGGATTACAGGC-3 and reverse, 5′-ACCTGAGGTCAGAAGTTCGAGAC-3′; (5) forward, 5′-GTCTCGAACTTCTGACCTCAGGT-3′ and reverse, 5′-CAGCTGGGCACTTATGAATATCCC-3′; (6) forward, 5′-GATATTGGTCTCCTTCGCCCTGT-3′ and reverse, 5′-GCAAGAGGAAGAATGACTGCTC-3′; and (7) forward, 5′-GAGCCTAACTGAAATCCCGCGA-3′ and reverse, 5′-CAAGCTCTGAGGACAGCCTCA-3. PCR products were visualized on a 2% agarose gel.

### Statistical Analysis

Data are expressed as mean ± SD. We performed a minimum of three independent experiments, where individual data points were based on at least technical duplicates each. Student's *t*-test was used for comparisons between two groups and ANOVA test to assess more than two groups. *P* < 0.05 were considered statistically significant. Statistical analysis of data was performed using GraphPad Prism 6.

## Results

### Gemcitabine Requires VMP1 Expression to Induce Autophagy in Pancreatic Cancer Cells Carrying Oncogenic *KRAS*

**Figure 1:**
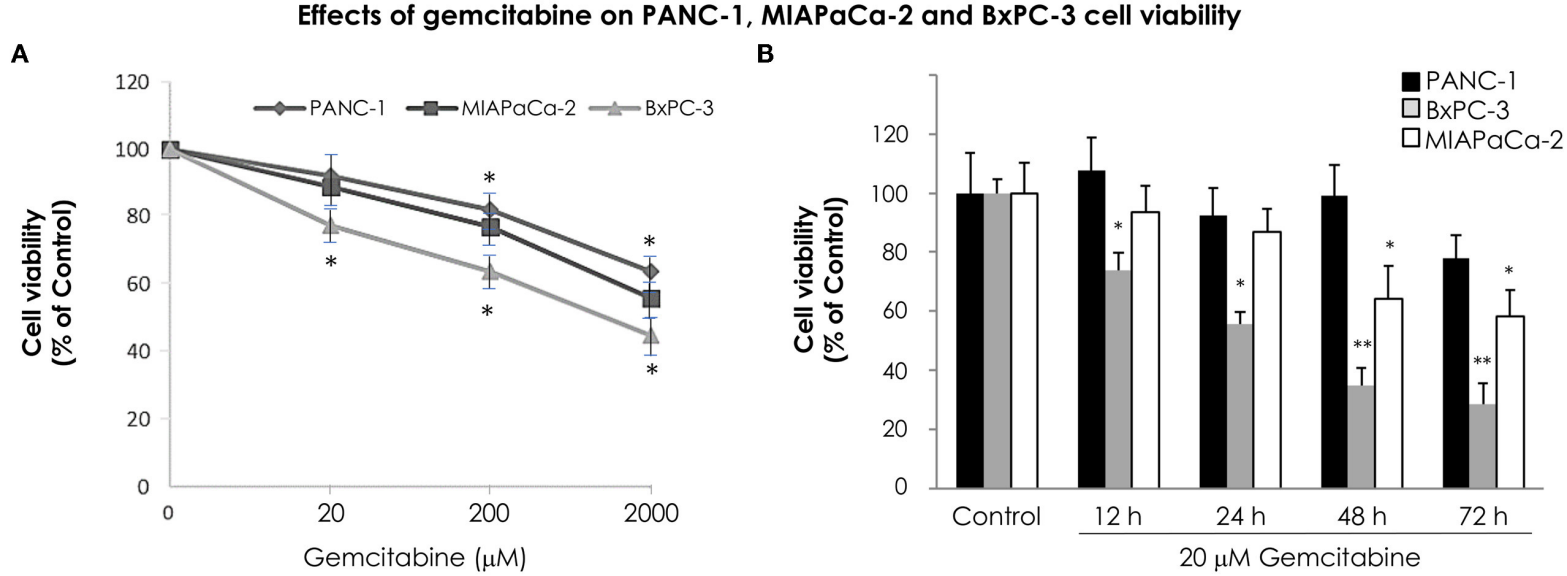
Relative sensitivity of pancreatic cancer cell lines PANC-1, MIAPaCa-2 and BxPC-3 to gemcitabine treatment. The number of viable cells was determined by the trypan blue dye exclusion test. **(A)** Cells were treated with increasing concentrations of gemcitabine (from 20 to 2,000 μM) for 24 h. BxPC-3 cells are sensitive to all gemcitabine concentrations tested, and PANC-1 and MIAPaCa-2 cells are sensitive from 200 μM gemcitabine. **(B)** Cells were treated with 20 μM gemcitabine and cell viability was determined at 0, 12, 24, 48, and 72 h. There were no significant differences within 72 h of treatment in PANC-1 cells, and within 48 h of treatment in MIAPaCa-2 cells. However, a significant reduction in cell viability was observed in BxPC-3 cells from 12 h of treatment. Results are presented as percentage of viable cells compared to the untreated control (mean ± SE; *n* = 3). *p < 0.05 vs. untreated cells, **p < 0.01 vs. untreated cells.

In order to analyze the time course effect of gemcitabine treatment on VMP1 expression we used PANC-1 and MIAPaCa-2 pancreatic tumor cells harboring a *KRAS* activating mutation, that are highly resistant to chemotherapy, and BxPC-3 pancreatic tumor cells that do not carry *KRAS* mutation. The relative sensitivity of pancreatic cancer cells to gemcitabine treatment was analyzed. PANC-1, MIAPaCa-2, and BxPC-3 cells were treated with 20, 200, and 2,000 μM gemcitabine. The relative number of viable cells was determined by the trypan blue dye exclusion test 24 h later. Figure 1A shows cell viability as a percentage relative to control according to the gemcitabine dose. BxPC-3 cells were more sensitive in all doses of gemcitabine analyzed (Figure 1A). However, cell viability was significantly reduced from 200 μM gemcitabine in PANC-1 and MIAPaCa-2 cells (Figure 1A). Thus, gemcitabine 20 μM is the lowest tested dose in which BxPC-3 cells are sensitive, and PANC-1 and MIAPaCa-2 are resistant, at least 24 h after treatment. In view of this, we analyzed cell viability of PANC-1, MIAPaCa-2, and BxPC-3 cells under 20 μM gemcitabine treatment during 72 h. There were no significant differences during the 72 h of treatment for PANC-1 cells and up to 48 h of treatment for MIAPaCa-2 cells. On the other hand, BxPC-3 cells showed a significant reduction in cell viability from 12 h of treatment (Figure 1B). These results indicate that at 12 and 24 h with 20 μM gemcitabine, PANC-1 and MIAPaCa-2 cells are resistant, and by comparison, BxPC-3 cells are sensitive to treatment. Consequently, from now on we use this dose of gemcitabine for the following experiments.

**Figure 2:**
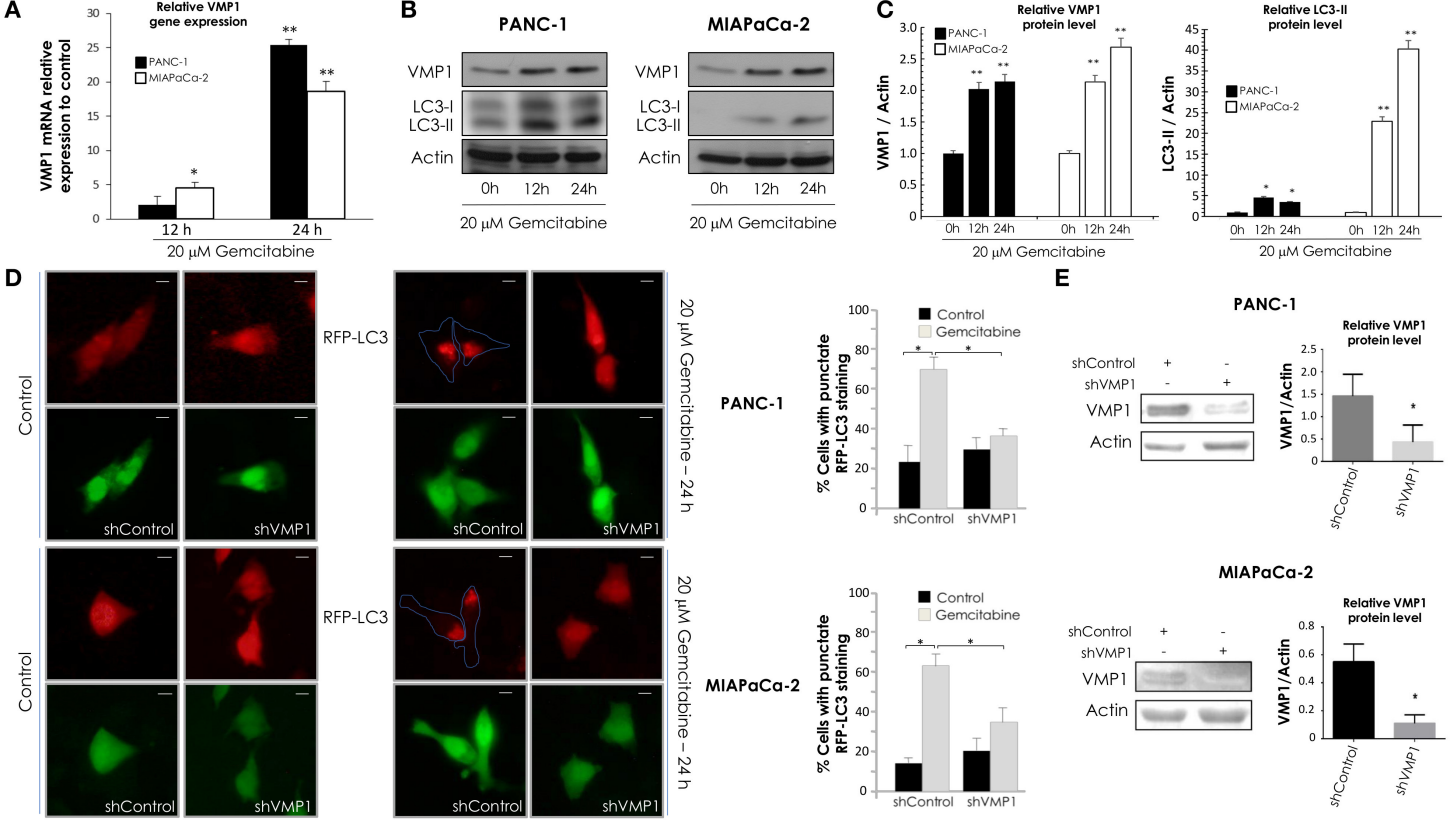
Gemcitabine requires VMP1 expression to induce autophagy in PANC-1 and MIAPaCa-2 cells. PANC-1 and MIAPaCa-2 cells were treated with 20 μM gemcitabine for 12 and 24 h. **(A)** qPCR and western blot analysis showed an increase of VMP1 mRNA **(A)** and protein expression **(B)** in PANC-1 and MIAPaCa-2 cells under treatment (*n* = 3). **(B)** Western blot analysis shows an increase of in PANC-1 and MIAPaCa-2 cells submitted to gemcitabine treatment. **(C)** Relative densitometry (VMP1/Actin and LC3-II/Actin) of western blots are shown in panel **(B)** (*n* = 3). **(D)** PANC-1 and MIAPaCa-2 cells were transiently co-transfected with RFP-LC3 and shVMP1-EGFP or shControl-EGFP expression vectors, and they were treated with 20 μM gemcitabine for 24 h. RFP-LC3 distribution was detected using fluorescence microscopy. Representative images show the punctate RFP-LC3 in cells expressing EGFP (bar scale = 10 μm). Quantitation represents the percentage of RFP-LC3 cells with punctate staining. LC3 redistribution was significantly reduced in shVMP1 expressing cells under gemcitabine treatment in both cell lines (*p < 0.05 vs. gemcitabine-treated shControl transfected cells). **(E)** PANC-1 and MIAPaCa-2 cells were transfected with the shControl or shVMP1 expression vectors. VMP1 expression was reduced by shVMP1 in PANC-1 and MIAPaCa-2 cells as was demonstrated by western blotting. Relative densitometry (VMP1/Actin) is shown (*n* = 3). *p < 0.05, **p < 0.01.

Next, PANC-1 and MIAPaCa-2 cells were incubated with 20 μM gemcitabine for 24 h and we evaluated VMP1 mRNA expression by qPCR assays. A significant induction of VMP1 mRNA was found after 12 and 24 h of gemcitabine treatment in PANC-1 and MIAPaCa-2 cells (Figure 2A). LC3 is currently used as a specific marker of autophagy (46). During the autophagic process, the cytosolic form of LC3 (LC3-I) undergoes C-terminal proteolytic and lipid modifications (LC3-II) and translocates from the cytosol to the autophagosomal membrane (47, 48). Then, we analyzed VMP1 expression and LC3 lipidation to determinate autophagy by western blot. Figure 2B shows that gemcitabine induced VMP1 protein expression and LC3-II formation, and therefore autophagy, in PANC-1 and MIAPaCa-2 cells. The quantification of western blots is shown in Figure 2C.

Afterward, PANC-1 and MIAPaCa-2 cells were co-transfected with RFP-LC3 and shVMP1 expression vectors and treated with 20 μM gemcitabine for 24 h. A diffuse pattern of LC3-RPF expression in the cytoplasm indicates that autophagy is not occurring, and when autophagy is induced RFP-LC3 is relocated as dots indicating the formation of autophagosomes. We counted cells with punctate RFP-LC3 staining to determinate autophagy (46). Figure 2D shows that gemcitabine induced RFP-LC3 redistribution to autophagosomes was significantly reduced when VMP1 expression was down-regulated in both cell lines. Figure 2E shows downregulation of VMP1 by shVMP1 expression in PANC-1 and MIAPaCa-2 cell lines. Therefore, gemcitabine requires VMP1 expression to induce autophagy in pancreatic tumor cells harboring a *KRAS* activating mutation.

### Gemcitabine Induces E2F1 Activation of VMP1-Mediated Autophagy Only in Pancreatic Tumor Cells That Carry Oncogenic *KRAS*

**Figure 3:**
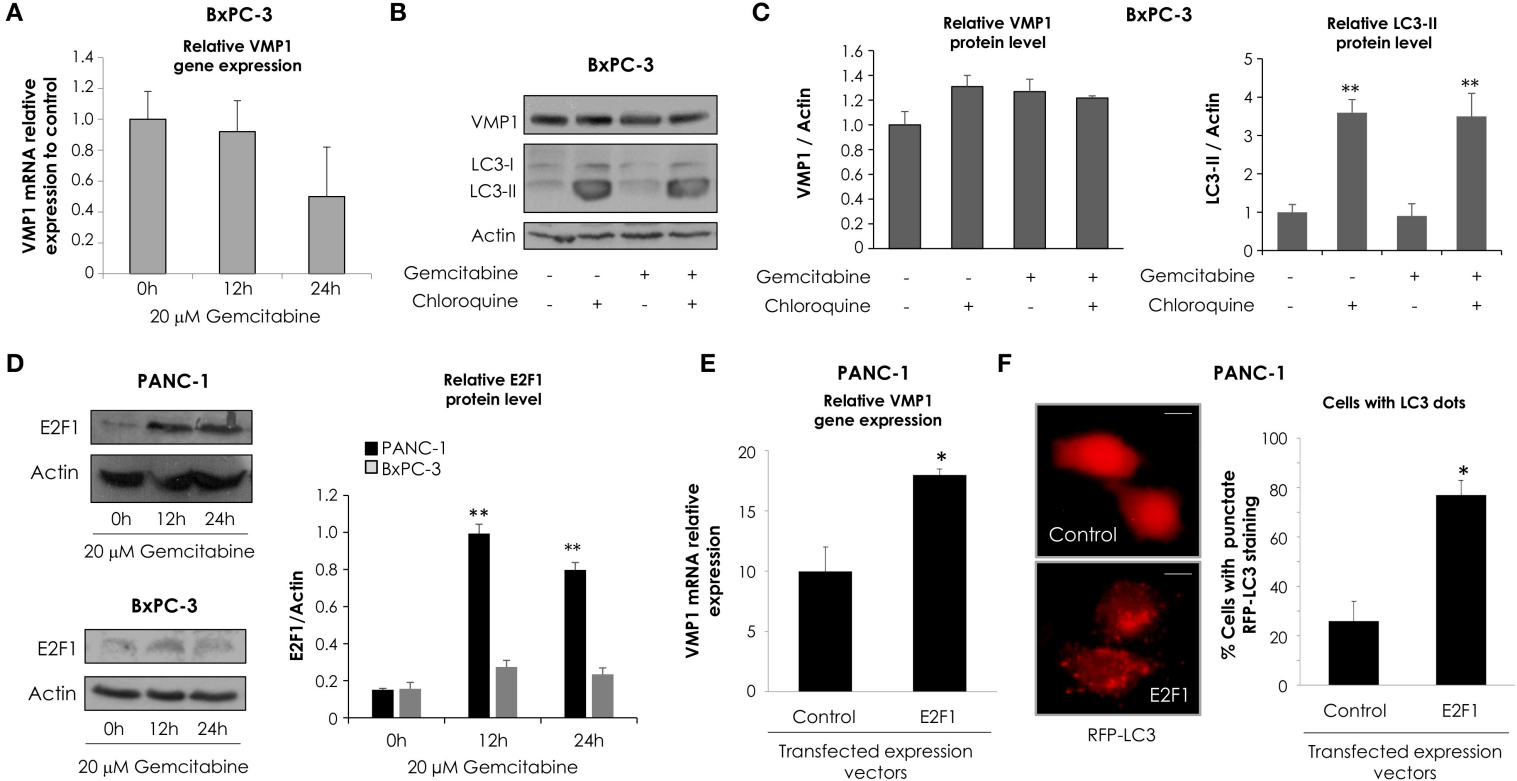
Gemcitabine induces E2F1 activation of VMP1-mediated autophagy only in pancreatic tumor cells that carry oncogenic KRAS. qPCR and western blot analysis showed no significant differences of VMP1 mRNA **(A)** and protein expression **(B)** in BxPC-3 treated with 20 μM gemcitabine for 12 and 24 h (*n* = 3). **(B)** Western blot analysis shows no difference of VMP1 expression and LC3 lipidation in BxPC-3 cells submitted to gemcitabine treatment. The use of chloroquine alone or in combination with gemcitabine treatment induced LC3-II accumulation compared to control cells or cells only treated with gemcitabine in the same proportion, respectively, indicating that gemcitabine does not interrupt autophagy flux in these cells. **(C)** Relative densitometry (VMP1/Actin and LC3-II/Actin) of western blots shown in B (*n* = 3). **(D)** Western blot analysis shows that E2F1 expression was increased in PANC-1 cells submitted to gemcitabine treatment for 12 and 24 h, and there were no significant differences in the expression of E2F1 in BxPC-3 cells. Relative densitometry (E2F1/Actin) of western blots is shown (*n* = 3). PANC-1 cells were transiently transfected with E2F1 expression vector or empty vector as control. **(E)** qPCR assay demonstrated an increase of VMP1 mRNA expression in PANC-1 cells expressing E2F1 compared to control cells (*n* = 3). **(F)** E2F1 expression induced autophagy as was determined by counting cells with punctate RFP-LC3 staining. Representative images of RFP-LC3 distribution using confocal microscopy are shown (bar scale = 10 μm). The graph presents the percentage of RFP-LC3 cells with punctate staining (*n* = 3). *p < 0.05, **p < 0.01.

Following, in order to analyze VMP1 expression and autophagy in cells that carry wild type *KRAS*, BxPC-3 cells were incubated with 20 μM gemcitabine for 24 h. Interestingly, gemcitabine treatment did not increase VMP1 mRNA and protein levels compared to basal conditions in BxPC-3 cells (Figures 3A,B). Then, we analyzed LC3 lipidation to determinate autophagy, and western blot analyses shows that gemcitabine treatment did not induce autophagy evidenced by LC3-II formation in BxPC-3 cells (Figure 3B). In addition, choloroquine treatment was used to evaluate autophagic process by inhibiting autophagy flux (46). The use of chloroquine alone or in combination with gemcitabine treatment induced LC3-II accumulation compared to control cells or cells only treated with gemcitabine in the same proportion, respectively, indicating that gemcitabine did not interrupt autophagy flux in these cells. The quantification of western blots is shown in Figure 3C. These results suggest BxPC-3 cells have a basal VMP1 expression and autophagy that are not up-regulated by gemcitabine.

E2F transcription factors are involved in cell proliferation and DNA repair (49). As gemcitabine incorporation into DNA is critical for its toxicity (50), we evaluated E2F1 expression in response to gemcitabine treatment. To characterize this response of E2F1 to gemcitabine, we chose the sensitive cell line BxPC-3 and the most resistant to treatment by comparison, PANC-1 cells. Western blot assay shows E2F1 protein levels were significantly increased after 12 and 24 h of gemcitabine treatment in PANC-1 cells, and they were similar with respect to control in treated BxPC-3 cells (Figure 3D). In consequence, E2F1 is a candidate to mediate increased VMP1 expression and autophagy in response to gemcitabine in PANC-1 but not in BxPC-3 cells.

Considering that VMP1 expression induces autophagosome formation (31), we tested whether E2F1 expression is capable of inducing VMP1 expression and autophagy in PANC-1 cells. We performed qPCR on samples from cells transfected with an expression vector for E2F1. As seen in Figure 3E, E2F1 expression induced VMP1 mRNA expression in PANC-1 tumor cells. Then, PANC-1 cells were concomitantly transfected with an expression plasmid encoding for the RFP-LC3 fusion protein and E2F1 expression vector. Figure 3F shows the recruitment of LC3 fusion protein in punctate structures in E2F1-transfected cells in contrast to the diffuse RFP-LC3 fusion protein signal observed in control cells. Quantitation showed that recruitment of LC3 was significantly increased in cells expressing E2F1 compared to control cells (Figure 3F). These results demonstrate that E2F1 is capable of inducing VMP1 expression and autophagy in pancreatic tumor cells resistant to gemcitabine treatment.

### *VMP1* Promoter Is Activated by Gemcitabine

**Figure 4:**
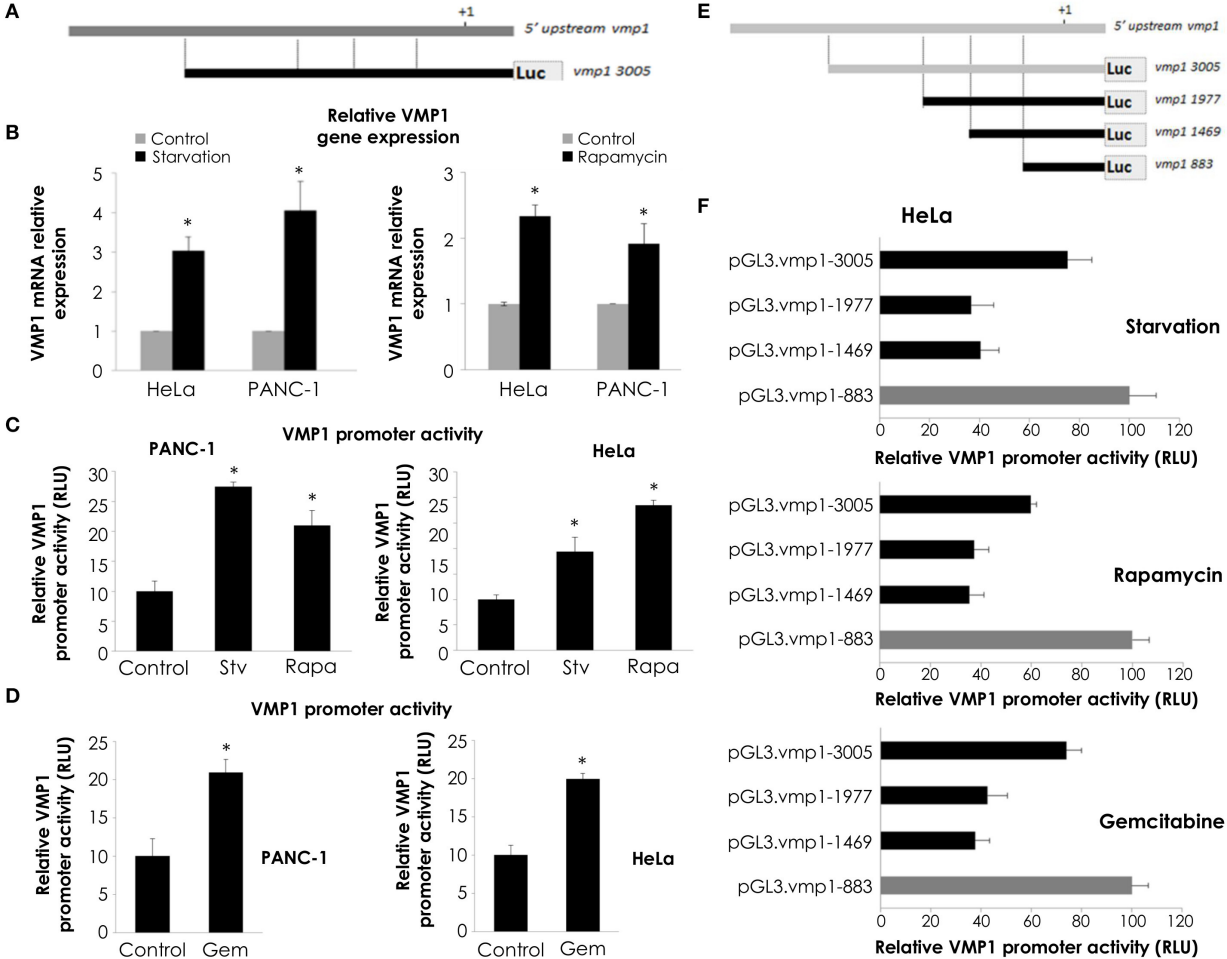
*VMP1* promoter analysis. **(A)** To assay *VMP1* promoter activity, a 3,005 bp sequence of the 5′ upstream region of the *VMP1* gene was amplified and cloned into the pGL3 reporter vector (pGL3.vmp1-3005) driving Luciferase (Luc) expression. **(B)** qPCR assay showed an increase of VMP1 mRNA expression in HeLa and PANC-1 cells submitted to starving conditions and rapamycin treatment for 4 h (*n* = 3). Next, HeLa and PANC-1 cells were transfected with pGL3.vmp1-3005. **(C)** Relative Luciferase activity shows an increase of *VMP1* promoter activation in HeLa and PANC-1 cells submitted to starving (Stv) conditions and rapamycin (Rapa) treatment. (*n* = 3). **(D)** Relative Luciferase activity shows an increase of *VMP1* promoter activation in HeLa and PANC-1 cells submitted to gemcitabine treatment for 24 h (*n* = 3). **(E)** Consecutive shorter fragments of 3005 bp sequence of *VMP1* promoter were amplified and subcloned in the pGL3 reporter vector (pGL3.vmp1-1977, pGL3.vmp1-1469, pGL3.vmp1-883). **(F)** HeLa cells were submitted to starvation conditions, rapamycin and gemcitabine treatment for 24 h. Relative Luciferase activity showed higher levels of *VMP1* promoter activation in the shorter construct (pGL3.vmp1-883) compared to 3,005 bp sequence of *VMP1* promoter in all treatments. *p < 0.05.

We have previously demonstrated that starving conditions and rapamycin treatment induce VMP1 expression in HeLa cells (31). On the other hand, VMP1 expression is activated in PANC-1 human tumor cells carrying mutated (G12D) KRAS (28). In order to study the molecular mechanism that regulates VMP1 expression in the context of gemcitabine induced-autophagy in pancreatic tumor cells, a 3,005 bp sequence of the 5′ upstream region of the human gene *VMP1* was amplified and cloned in the pGL3 reporter vector (pGL3.vmp1-3005) (Figure 4A). Following, we analyzed if this sequence cloned has a promoter activity. In these experiments, we used HeLa cells under starving conditions and rapamycin treatment as a positive control of autophagy induction, and the pGL3.vmp1-3005 construct was used to perform luciferase reporter assays. As a result, we found increased VMP1 mRNA expression and *VMP1* promoter activity in response to starving conditions and to rapamycin treatment in PANC-1 and HeLa cells (Figures 4B,C). Next, we analyzed if gemcitabine was able to increase VMP1 promoter activity. Figure 4D shows that the activity of the 3,005 bp sequence of *VMP1* promoter was significantly increased when PANC-1 and HeLa cells were treated with 20 μM gemcitabine for 24 h.

In order to localize the essential promoter sequence in the 3,005 bp sequence of the 5′ upstream region of the human gene *VMP1*, we amplified and subcloned consecutive shorter fragments of this region. Three more constructs were created and named as follows: pGL3.vmp1-1977, pGL3.vmp1-1469, and pGL3.vmp1-883 (Figure 4E). Luciferase activity assays were performed in HeLa cells transfected with each construct and submitted to starvation, rapamycin, or gemcitabine treatment. Relative promoter activity was analyzed for each sequence (Figure 4F). Results showed a decreased activation for the pGL3.vmp1-1977 and pGL3.vmp1-1469 constructs comparing to the initial 3,005 bp sequence. On the other hand, the activity was increased when the shorter pGL3.vmp1-883bp construct was used. The same results were observed in all the conditions analyzed. These data suggest that essential regulation motifs involved in VMP1 expression are contained into this *VMP1* shorter promoter region of 883 bp.

### E2F1 Directly Activates the *VMP1* Promoter and Regulates VMP1 Expression Under Gemcitabine Treatment

**Figure 5:**
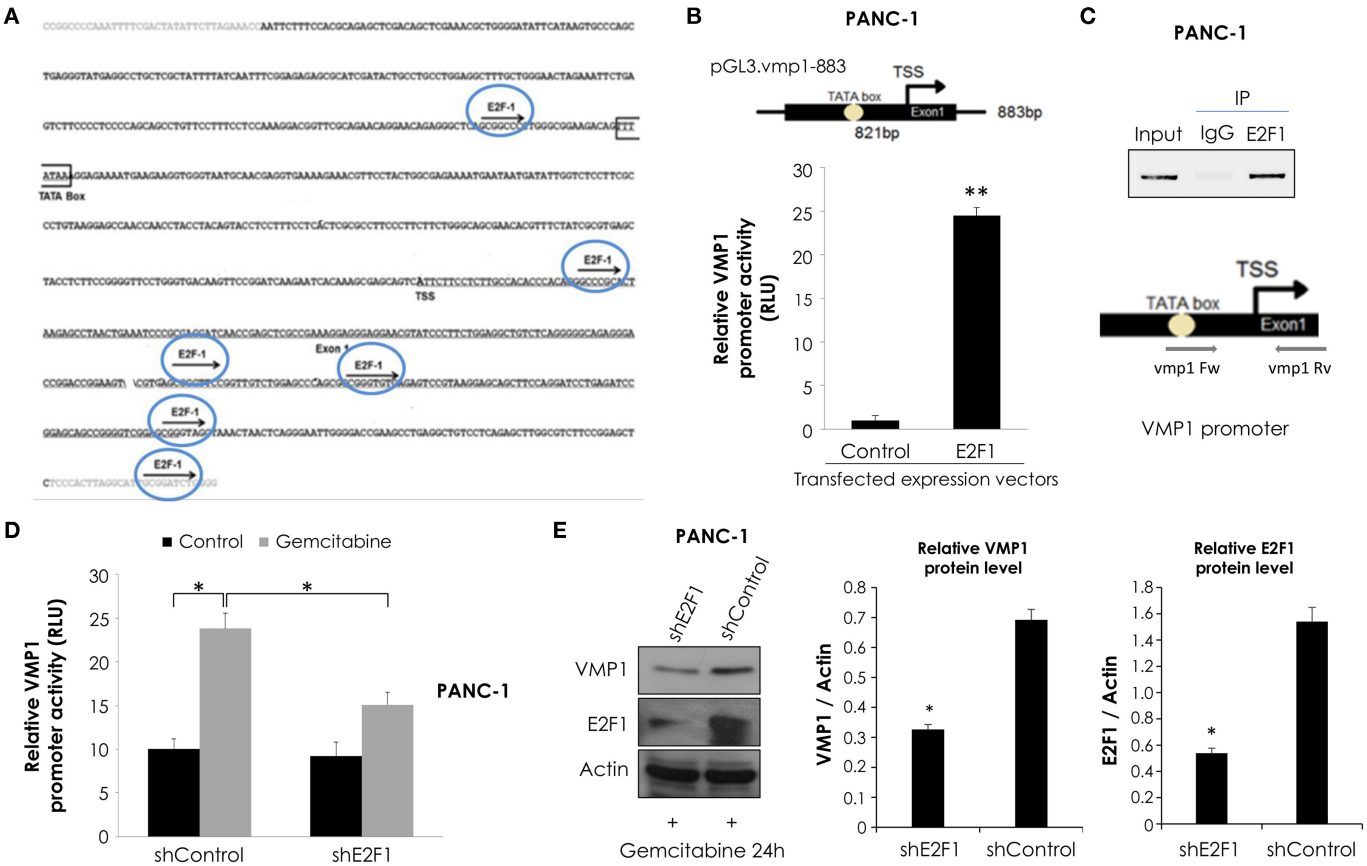
E2F1 binds to *VMP1* promoter and regulates VMP1 expression induced by gemcitabine. **(A)** Bioinformatics analysis of the *VMP1* promoter identified candidate E2F1 binding sites. *TSS*, transcription starting site. **(B)** PANC-1 cells were transiently transfected with pGL3.vmp1-883, E2F1 expression vector or empty vector as control. Relative luciferase activity showed an increase of *VMP1* promoter activation in PANC-1 cells expressing E2F1 compared to control (*n* = 3). **(C)** ChIP assay using chromatin extracts precipitated with an antibody against E2F1 or IgG as a negative control in PANC-1 cells treated with 20 μM gemcitabine for 24 h. Through PCR we amplified the region of *VMP1* promoter in anti-E2F1 precipitated products using a specific set of primers (vmp1 Fw and vmp1 Rv) as described in Experimental Procedures, indicating that E2F1 binds directly to the *VMP1* promoter. **(D)** PANC-1 cells were transiently transfected with shControl or shE2F1 expression vectors and treated with 20 μM gemcitabine for 24 h. Relative Luciferase activity showed a significant reduction of *VMP1* promoter activation in PANC-1 cells expressing shE2F1 and treated with 20 μM gemcitabine for 24 h (*n* = 3). **(E)** Western blot analysis showed reduction of VMP1 expression in PANC-1 cells transfected with shE2F1 targeting vector and treated with 20 μM gemcitabine for 24 h. Relative densitometries (VMP1/Actin and E2F1/Actin) of western blots are shown (*n* = 3). *p < 0.05, **p < 0.01.

Considering that maximal promoter activity in luciferase reporter assays was observed for the pGL3.vmp1-883 construct, we analyzed this sequence using bioinformatics tools. This *in silico* analysis data showed a putative promoter in this sequence containing a TATA. In addition, we identified several putative binding sites for relevant transcription factors related to the cellular stress response including E2F1 (Figure 5A).

In order to know if *VMP1* gene is a direct target of E2F1, we used a combination of transcriptional and chromatin assays to determine a possible involvement of E2F1 transcription factor in *VMP1* promoter activation. First, we performed luciferase reporter assays using the shorter sequence of VMP1 promoter, pGL3.vmp1-883bp construct. Reporter studies demonstrate that expression of E2F1 led to an increase in *VMP1* promoter activity (Figure 5B). Moreover, endogenous E2F1 can bind to *VMP1* promoter in PANC-1 cells treated with gemcitabine as demonstrated by ChIP assay (Figure 5C). Also, gemcitabine increased VMP1 promoter activity (see shControl transfected PANC-1 cells) (Figure 5D). Moreover, down-regulation of E2F1 expression in PANC-1 cells treated with gemcitabine significantly reduced VMP1 promoter activity (Figure 5D) and VMP1 expression (Figure 5E). These results demonstrate that VMP1 is a novel direct target of the E2F1 transcription factor under gemcitabine treatment.

### E2F1 and EP300 Cooperate in *VMP1* Promoter Activation

**Figure 6:**
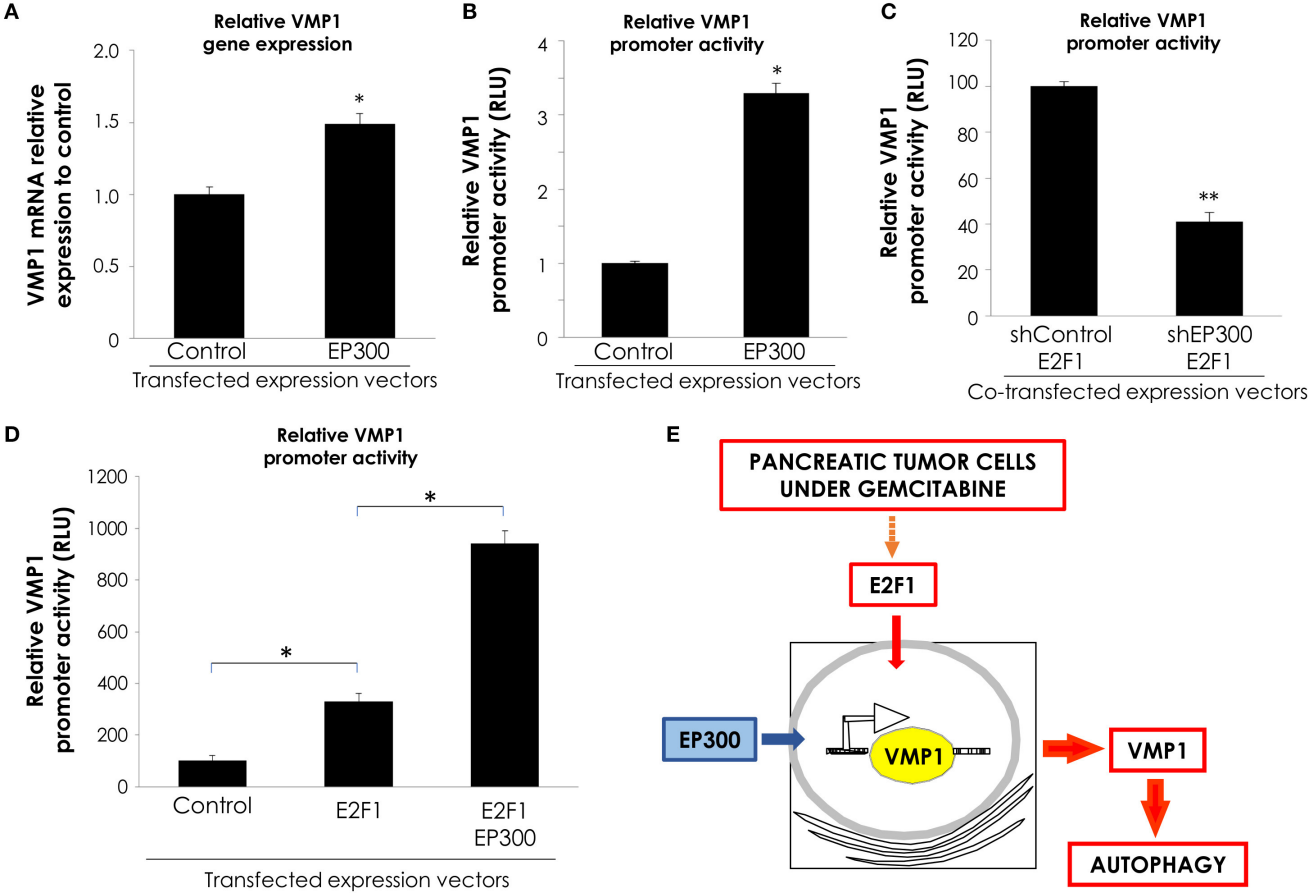
E2F1 and EP300 cooperates in *VMP1* promoter regulation. PANC-1 cells were transiently transfected with an EP300 expression vector or with and empty expression vector. **(A)** VMP1 mRNA increased expression was observed by qPCR. In PANC-1 transfected with an EP300 expression vector compared to control cells (*n* = 3). **(B)** PANC-1 cells were transiently transfected with pGL3.vmp1-883. Relative Luciferase activity showed a significant increase of *VMP1* promoter activation in PANC-1 cells co-transfected with EP300 expression vector compared to cells transfected with a control vector (*n* = 3). **(C)** PANC-1 cells were transiently co-transfected with pGL3.vmp1-883, an E2F1 expression vector and shEP300 targeting vector or shControl targeting vector. Relative Luciferase activity showed a reduction of *VMP1* promoter activity in PANC-1 cells co-transfected with E2F1 and shp300 targeting vector compared to cells transfected with a E2F1 and shControl targeting vector (*n* = 3). **(D)** PANC-1 cells were transiently co-transfected with pGL3.vmp1-883, E2F1, and EP300 expression vectors. Relative Luciferase activity showed an increase of *VMP1* promoter activation in PANC-1 cells co-transfected with E2F1 and EP300 expression vectors compared to cells transfected with a E2F1 expression vector alone (*n* = 3). **(E)** Schematic model. E2F1-p300-VMP1 pathway that mediates gemcitabine-induced autophagy in pancreatic cancer cells. *p < 0.05, **p < 0.01.

Transcription factors regulate gene expression through their inherent activation or repression properties, and through functional interactions with co-regulatory molecules. Here, we tested whether activation by E2F1 involves the histone acetyltransferases EP300. First, we analyzed if EP300 could induce VMP1 mRNA expression in PANC-1 cells. Figure 6A, shows that VMP1 mRNA expression was up-regulated in PANC-1 cells expressing EP300. Additionally, EP300 expression significantly activated the *VMP1* promoter compared to control cells (Figure 6B). Then, we evaluated if EP300 participates with E2F1 in *VMP1* promoter activation. Interestingly, down-regulation of EP300 impaired E2F1-mediated activation of the *VMP1* promoter in PANC-1 cells (Figure 6C). Furthermore, co-expression of E2F1 and EP300 led to a synergistic activation of the VMP1 promoter (Figure 6D). These findings demonstrate that E2F1 and EP300 cooperates in *VMP1* promoter regulation.

Altogether, these data show that the E2F1-EP300 activator/co-activator complex is part of the novel signaling pathway controlling the promoter activity and, consequently, the expression of the autophagy-related gene VMP1 in pancreatic tumor cells carrying oncogenic *KRAS*.

## Discussion

Tumor cells with a high prevalence of *KRAS* activating mutations, like pancreatic cancer, have the distinction of a poor prognosis (4). Previously, it has been demonstrated that many human cancer cell lines with *KRAS* activating mutations have basal levels of autophagy (51, 52). Yang et al. (6), have showed that pancreatic cancer cells exhibit constitutive autophagy under basal conditions, and it is increased in the advanced stages of PDAC being required for malignant transformation. In a previous work, we have identified VMP1 as a transcriptional target of oncogenic KRAS signaling pathway and demonstrated that KRAS requires VMP1 to induce and maintain basal autophagy in pancreatic tumor cells (28). On the other hand, VMP1-mediated autophagy is early induced above basal conditions by gemcitabine treatment in MIAPaCa-2 cells (36). Moreover, VMP1 is highly expressed in poorly differentiated human pancreatic cancer (41). In this study, we identified a regulatory pathway, which is activated by gemcitabine treatment, in pancreatic tumor cells carrying a KRAS mutation at amino acid position 12. This molecular mechanism involves a novel E2F1-EP300-VMP1 pathway controlling VMP1 expression triggered by gemcitabine.

According to the *Ensembl Genome database, VMP1* gene is localized in Chromosome 17 of the human genome. Using informatics tools, we found out four putative promoter regions given for *VMP1* gene; among them we particularly focus on one of 821 bp localized in the positive strand at the following position 57784363–57785183 of chromosome 17. In the analysis, it was also found the proposed transcription-starting site (TSS) (Figure 5A) at position 501 of the promoter area. This prediction would regulate the expression of a 12 exons transcript and we consider this transcript to be the one coding for the 406 aa protein VMP1. The TSS is located at the start of the first exon sequence, while the starting codon (ATG) is in the second exon. Considering these data, we amplified and cloned a 3,005 bp sequence of the 5′ upstream regions of the *VMP1* gene sequence which includes the promoter area analyzed above. Using luciferase reporter assays we demonstrated that this region is activated by previously reported VMP1 stimuli, such as rapamycin, starvation, and gemcitabine (Figure 4). We found that regulatory motifs involved in VMP1 expression are contained into an 883 bp fragment in the 5′ upstream region of the *VMP1* gene. This essential promoter sequence showed the highest activation by rapamycin, starvation, and gemcitabine treatment using luciferase reporter assays.

The *in silico* analysis of VMP1 essential promoter sequence revealed the presence of binding sites for several transcription factors related to cellular stress. We focus our attention on E2F1 because it has been correlated with high-grade tumors and unfavorable patient survival in PDAC (53, 54). E2F1 was the first identified member of the E2F family of transcription factors. E2F activity is linked to retinoblastoma tumor suppressor (RB)–dependent cell-cycle control. E2F transcription factors are found downstream of growth factor signaling cascades, acting as transcriptional activators or repressors of genes necessary for cell cycle progression (55). Most human tumors harbor the functionally inactivated retinoblastoma protein, resulting in deregulated E2F1 and its target genes are highly up-regulated in these transformed cells (56). This up-regulation leads to the activation of cytoplasmic (PIK3CA/AKT and RAS/MAPK/ERK) and nuclear signaling cascades related to invasion and metastasis (54). Also, activation of E2F1 transcription factor has been shown to induce autophagy (57) by up-regulating the expression of the autophagy genes LC3, ULK1 (unc-51 like autophagy activating kinase 1), ATG5 and DRAM1 (DNA damage regulated autophagy modulator 1). The E2F1-mediated induction of LC3, ULK1, and DRAM1 is direct (through interaction with the promoter), whereas the up-regulation of the expression of ATG5 is indirect (58). In this work, we provide evidence of another gene related to autophagy that is up-regulated by E2F1. We demonstrated that E2F1 is able to induce autophagy in pancreatic tumor cells and regulates VMP1-mediated autophagy by a direct binding to VMP1 promoter.

Gemcitabine inhibits DNA synthesis via a process called masked chain termination where gemcitabine is incorporated into DNA via DNA polymerase α, leading to the inhibition of DNA repair and synthesis (59). It has been shown that E2F1 induces genes involved in DNA repair in normal cells and in tumor cells undergoing chemotherapy through complex formation on the promoters of these genes (54). In this sense, BxPC-3 cells that are sensitive to the dose of 20 μM gemcitabine did not increase the expression of E2F1 or VMP1 during treatment. In contrast, PANC-1 cells resistant to treatment increased the expression of E2F1, result consistent with data of previous works (60, 61). Lai et al. (60) have demonstrated that PANC-1 cells respond to gemcitabine by increasing the expression of ribonucleotide reductase M2 catalytic subunit (RRM2) through E2F1-mediated transcriptional activation, as a DNA damage response to enhance DNA repair capacity in these cells. Here, we demonstrated E2F1 and VMP1 expressions are both increased in PANC-1 cells treated with gemcitabine. Besides, pancreatic tumor cells transfected with an expression vector for E2F1 induced VMP1 expression and activated autophagy. Therefore, in this study we demonstrated another mechanism activated by E2F1 in response to chemotherapy, in which E2F1 activates VMP1 expression and autophagy as a resistance response to gemcitabine.

Autophagy is constitutively activated in oncogenic KRAS-driven tumors and is necessary for the development of these tumors (6). Previously, we identified the PI3KCA (phosphatidylinositol-4,5-biphosphate 3-kinase catalytic subunit alpha)-AKT1 (AKT serine/threonine kinase 1) pathway as the signaling pathway mediating the expression and promoter activity of VMP1 in KRAS driven tumors (28). PANC-1 cells harbor a *KRAS* mutated allele (KRAS G12D) and these cells present basal expression of VMP1 and autophagy. In this work, we show that gemcitabine treatment increased that basal VMP1 expression and autophagy in PANC-1 cells. As well as GLI3 (GLI family zinc finger 3) is the transcription factor implicated in *VMP1* promoter activation in basal conditions in PANC-1 cells (28), E2F1 plays that function under gemcitabine treatment. Here, we show that down-regulation of E2F1 reduced VMP1 expression and *VMP1* promoter activity induced by gemcitabine. Besides, we demonstrated a direct binding of E2F1 on *VMP1* promoter under gemcitabine treatment in pancreatic tumor cells. Therefore, these data suggest VMP1 expression is regulated by different transcription factors depending on the cellular context that autophagy is induced. Further research will be necessary to clarify if function or mechanisms involved in basal VMP1-induced autophagy differs from VMP1-induced autophagy under gemcitabine treatment.

The regulation of gene expression depends on the characteristic activation/repression properties of each transcription factor, but also on functional interactions with co-regulatory molecules. EP300 belongs to the type 3 family of lysine acetyltransferases (KAT3) (62), and this enzyme is involved in the regulation of important physiological processes such as proliferation, differentiation, and apoptosis, due to its ability to function as transcriptional coactivator interacting and regulating more than 400 transcription factors (63, 64). However, the role of EP300 in gene regulation is not only restricted to its property of allowing the binding of transcription factors to large protein complexes in the transcription machinery, but also implies the required KAT activity for the acetylation of transcription factors and histones that allow access to chromatin (65). Thus, EP300 contributes to DNA repair through histone acetylation, facilitating the recruitment of DNA repair factors to the site of damage (66). We demonstrated that EP300 potentiates the *VMP1* promoter activation by E2F1. Even, down-regulation of EP300 impaired E2F1-mediated activation of the VMP1 promoter in pancreatic tumor cells. These findings demonstrate that E2F1 and p300 cooperate in VMP1 promoter regulation.

Our results agree with Hashimoto et al. (67), who have shown that autophagy has a cytoprotective effect against 5-fluorouracil and gemcitabine in pancreatic cancer cells. They demonstrated that inhibition of autophagy potentiates the inhibition of PANC-1 cell proliferation by 5-fluorouracil and gemcitabine. Here, we showed an induction of VMP1 expression and autophagy in PANC-1 and MIAPaCa-2 cells under gemcitabine treatment and down-regulation of VMP1 expression significantly reduced autophagy induced by gemcitabine. These data strongly suggest that VMP1 expression is involved in PDAC chemoresistance to gemcitabine.

In conclusion, we have identified the E2F1-EP300-VMP1 pathway that mediates gemcitabine-induced autophagy in pancreatic cancer cells (Figure 6E). This pathway would be activated by gemcitabine like a resistance mechanism. Our results point at E2F1 as a regulatory factor modulating gemcitabine induced VMP1-mediated autophagy in human pancreatic cancer cells and mechanistically integrate the autophagic degradative process into the complex network of events involved in PDAC chemoresistance.

## Data Availability Statement

The datasets generated for this study are available on request to the corresponding author.

## Author Contributions

AR designed and performed experiments, analyzed and interpreted data, prepared figures, and wrote the manuscript. CC performed experiments, contributed to interpret data, and prepared figures. FR performed experiments. VB developed analytical tools. TO developed expression vectors. CG contributed to interpret data. MV developed the hypothesis, designed experiments, analyzed, and interpreted data, and wrote the manuscript. All authors reviewed the manuscript.

## Conflict of Interest

The authors declare that the research was conducted in the absence of any commercial or financial relationships that could be construed as a potential conflict of interest.

## Abbreviations

ATG: related to autophagy
ChIP: chromatin immunoprecipitation
E2F1: E2F transcription factor 1
EGFP: enhanced green fluorescent protein
EP300: E1A binding protein p300
HIF1A: hypoxia inducible factor 1 subunit alpha
KRAS: Kirsten rat sarcoma viral oncogene homolog
LC3: microtubule-associated protein 1 light chain 3
PDAC: pancreatic ductal adenocarcinoma
PBS: phosphate-buffered saline
RLU: Relative light units
RFP: red fluorescent protein
shControl: short hairpin RNA with a scrambled sequence
shE2F1: short hairpin RNA targeting E2F1
shEP300: short hairpin RNA targeting EP300
shVMP1: short hairpin RNA targeting VMP1
TBS: TRIS-buffered saline
TTS: transcriptional starting site
VMP1: Vacuole Membrane Protein 1.
